# A rare case of a patient with cystinosis and COVID-19 pneumonia with difficult weaning from mechanical ventilation: the “pocus force”

**DOI:** 10.1186/s44158-022-00053-8

**Published:** 2022-06-13

**Authors:** Luigi Vetrugno, Valentina Angelini, Simone Antonio Smiraglia, Elisabetta Saraceni, Pierluigi Di Giannatale, Salvatore Maurizio Maggiore

**Affiliations:** 1 Department of Medical, Oral and Biotechnological Sciences, University of Chieti-Pescara, Chieti, Italy; 2 Department of Anesthesiology, Critical Care Medicine and Emergency, SS. Annunziata Hospital, Via dei Vestini, 66100 Chieti, Italy; 3 Department of Innovative Technologies in Medicine and Dentistry, Gabriele d’Annunzio University of Chieti-Pescara, Chieti, Italy

**Keywords:** Mechanical ventilation, Weaning, Diaphragmatic ultrasound, Cystinosis, COVID-19

## Abstract

Here, we describe the case of a 39-year-old woman with cystinosis who already suffered from an extra parenchymal pattern of restrictive lung disease and, after SARS-CoV-2-related respiratory failure, had a difficult weaning from mechanical ventilation and required tracheostomy. In this rare disease, due to the mutation of the CTNS-gene located on chromosome 17p13, cystine accumulation in the distal muscle has been reported, even in the absence of manifest muscle fatigue. We were able to evaluate diaphragmatic weakness in this patient through the ultrasonographic evaluation of the diaphragm. We believe that diaphragm ultrasonography could be helpful to identify causes of difficult weaning thus supporting clinical decisions.

**Supplementary Information:**

The online version contains supplementary material available at 10.1186/s44158-022-00053-8.

## Case presentation

**Fig. 1:**
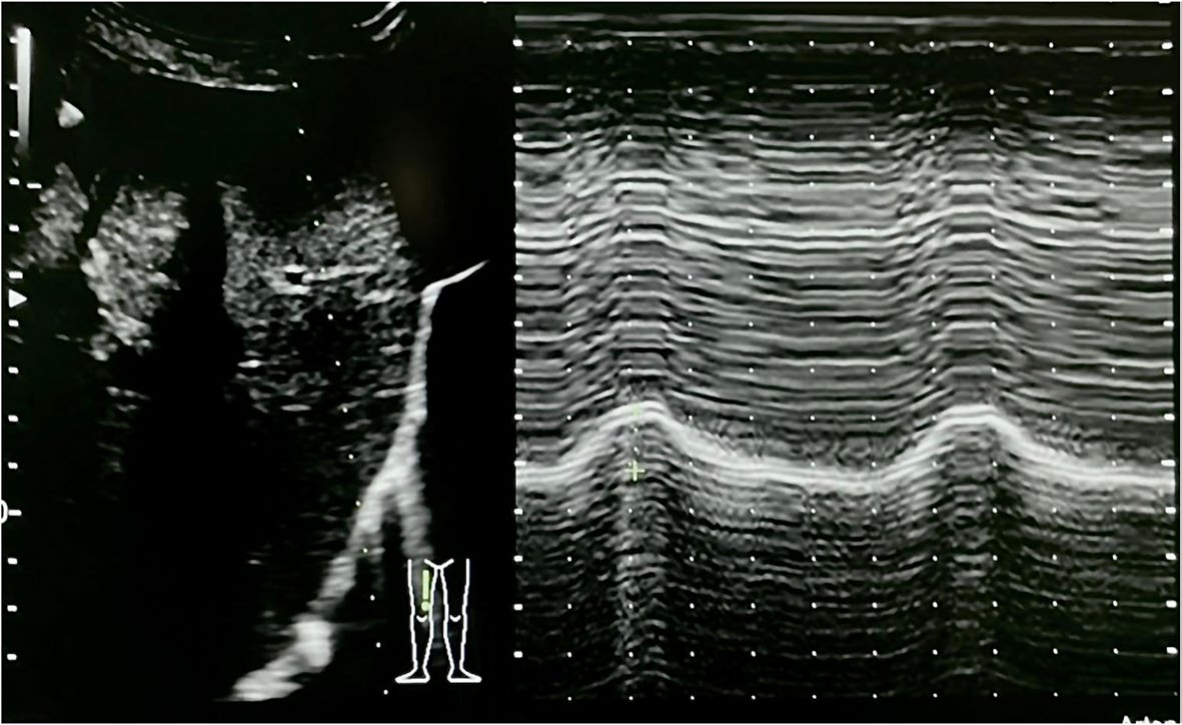
A low-frequency (3.5–5 MHz) ultrasound transducer is used to identify the right hemidiaphragm. The M-mode then is employed to show movements and measure diaphragmatic excursion (cm)

A 39-year-old vaccinated woman was admitted to our emergency department with dyspnea and respiratory failure with a positive nasopharyngeal molecular swab test for SARS-CoV-2 infection. After 6 days of O_2_ therapy, the patient was transferred to the intensive care for respiratory worsening requiring non-invasive ventilation (NIV) with a helmet (FiO_2_ 60%, PS 8 cmH_2_O, PEEP 12 cmH_2_O). For the further 6 days, the patient alternated NIV and high flow nasal oxygen (HFNO) (FiO_2_ 50%, flow 60 L/min) until, after an episode of desaturation and respiratory distress with visual analogic scale (VAS) for dyspnea of 6 (on a 0-10 scale), required tracheal intubation, and she was transferred to intensive care for invasive mechanical ventilation. The respiratory gas exchange and chest x-ray slowly improved in the following 7 days (arterial blood gas on the 7th day: pH 7.38, PaO_2_ 231 mmHg, PaCO_2_ 47 mmHg, HCO_3_ 28.1 mmol/L, BE 2.6 mmol/L, Lactate 1.20 mmol/L), and we attempted to wean the patient from the ventilator reducing the pressure support to 8 cmH_2_0 with 5 cmH_2_O of PEEP, however, without success. Clinically, the patient showed increased accessory respiratory muscles’ fatigue, and the PaCO_2_ increased > 100 mmHg; tidal volume (Vt) was less than 300 mL with respiratory rate (RR) of 26/min. After 2 unsuccessful attempts of reducing pressure support, she was tracheostomized [1, 2]. Just after the second weaning attempt and before performing the tracheostomy, point-of-care ultrasonography (POCUS) with low-frequency probe (Hitachi, Arietta 65, Tokyo 110-0015 Japan) revealed a lung ultrasound score (LUS) of 8 and with high-frequency probe diaphragmatic weakness with a thickening fraction (TF) < 20% (normal value between 20 and 30%). TF was measured as the maximal diaphragm thickness during inspiration (Tdi, pi) minus the diaphragm thickness at end-expiration (Tdi, ee) divided by the Tdi, ee, and multiplied by 100. Ultrasonographic diaphragm assessment was performed while the patient was ventilated with pressure support ventilation of 8 cmH_2_0 and 5 cmH_2_O of PEEP; in the supine position, the diaphragmatic excursion was < 1.3 cm (normal value > 1.8 cm) [3] (Fig. 1 and supplemental video 1). Supplemental video 2 showed TF measurement.

## Patient history

The patient’s past medical history was characterized by cystinosis, type II diabetes mellitus (DM), recurrent urinary tract infections, hyperthyroidism, and previous double kidney transplantations (in 1998 and 2016). She suffered from an extra parenchymal pattern of restrictive lung disease, with intermittent O_2_ therapy. The patient was under treatment with the following pharmacology therapy: steroids, L-thyroxin, mycophenolic acid, proton pump inhibitor, linagliptin, bisoprolol, tacrolimus, and cholecalciferol.

## Clinical note

Cystinosis is a rare autosomal recessive lysosomal storage disorder due to the mutation of the CTNS-gene located on chromosome 17p13, which codes for cystinosis. Three forms have been described (infantile, juvenile, ocular), depending on the symptoms’ onset and severity. The defective cystinosin protein accumulates and crystallizes cystine in the lysosome, but how this causes tissue damage and leads to the typical clinical symptoms is not well understood [4]. Deposit of cystine in various organs causes hyperthyroidism, insulin-dependent diabetes, hepatosplenomegaly with portal hypertension, and muscle and brain involvement. The disease rapidly evolves toward kidney failure. The accumulation leads to retinal blindness and posterior ocular synechiae, diabetes mellitus, infertility in males due to primary hypogonadism, encephalopathy with confusion, memory loss, and cerebral atrophy of distal myopathy [5]. With the patient growing, cysteamine reduces Cristina’s leukocyte concentration, slowing the evolution towards renal failure. However, the disease did not recur after kidney transplantation. Distal myopathy has been described as cystine accumulation in the distal muscle, even in the absence of manifest muscle weakness. Swallowing muscles can be involved, and dysphagia is quite common. Cystinosis myopathy is a lysosomal disease that involves the skeletal muscles. The lysosomal involvement of the muscle tissue is confirmed by the presence of autophagic acid phosphatase positive vacuoles which are the pathological hallmark of cystinosis myopathy [6].

## Clinical message

Searching on PubMed, we found only a case report on diaphragm myopathy in a cystinosis patient presenting hypoventilation successfully treated by nocturnal NIV [7]. In our case, the patient suffered from a neglected, mild respiratory muscle myopathy worsened due to COVID-19 pneumonia. We successfully identified diaphragmatic weakness using ultrasound during difficult weaning from mechanical ventilation in this patient, with diaphragmatic myopathy secondary to cystinosis syndrome. Ultrasound evaluation of the diaphragm helped us to identify the cause of difficult weaning and to orient the clinical decision to perform an early tracheotomy. Together with other parameters, diaphragm ultrasound could be helpful during weaning from mechanical ventilation, because it can predict extubation outcome, thus reducing unnecessarily prolonged intubations, and prevents emergent reintubations, with high sensitivity and specificity [8].

## Supplementary Information

**Additional file 1: Supplemental video 1.** Ultrasonographic diaphragm assessment.

**Additional file 2: Supplemental video 2.** TF measurement.

## Data Availability

The authors declare that the datasets used and/or analyzed during the current study are available from the corresponding author on reasonable request.
